# Downregulation of CD94/NKG2A inhibitory receptors on CD8^+ ^T cells in HIV infection is more pronounced in subjects with detected viral load than in their aviraemic counterparts

**DOI:** 10.1186/1742-4690-4-72

**Published:** 2007-10-10

**Authors:** Mustapha Zeddou, Souad Rahmouni, Arnaud Vandamme, Nathalie Jacobs, Frédéric Frippiat, Philippe Leonard, Nicole Schaaf-Lafontaine, Dolores Vaira, Jacques Boniver, Michel Moutschen

**Affiliations:** 1 University of Liège, Laboratory of Immunology and Infectious Diseases, GIGA-R, Liège, Belgium; 2 University of Liège, Department of Pathology, CRCE-GIGA, Liège, Belgium; 3 University of Liège, Department of Infectious Diseases, CHU, Liège, Belgium; 4 University of Liège, Department of Biological Haematology, CHU, Liège, Belgium

## Abstract

The CD94/NKG2A heterodimer is a natural killer receptor (NKR), which inhibits cell-mediated cytotoxicity upon interaction with MHC class I gene products. It is expressed by NK cells and by a small fraction of activated CD8^+ ^T lymphocytes. Abnormal upregulation of the CD94/NKG2A inhibitory NKR on cytotoxic T cells (CTLs) could be responsible for a failure of immunosurveillance in cancer or HIV infection. In this study, CD94/NKG2A receptor expression on CD8^+ ^T lymphocytes and NK cells was assessed in 46 HIV-1-infected patients (24 viraemic, 22 aviraemic) and 10 healthy volunteers. The percentage of CD8^+ ^T lymphocytes expressing the CD94/NKG2A inhibitory heterodimer was very significantly decreased in HIV-1-infected patients in comparison with non-infected controls. Within the HIV infected patients, the proportion of CD8^+ ^T lymphocytes and NK cells expressing CD94/NKG2A was higher in subjects with undetectable viral loads in comparison with their viraemic counterparts. No significant difference was detected in the proportion of CD8^+ ^T lymphocytes expressing the activatory CD94/NKG2C heterodimer between the HIV-1 infected patients and the healthy donors, nor between the vireamic and avireamic HIV-1 infected patients. In conclusion, chronic stimulation with HIV antigens in viraemic patients leads to a decreased rather than increased CD94/NKG2A expression on CD8^+ ^T lymphocytes and NK cells.

## Findings

The CD94/NKG2 heterodimer is a C-type lectin receptor, formed by the covalent association of CD94, a protein with a short non-signaling intracytoplasmic tail [1], and one of the NKG2 molecules. To generate a functional receptor, CD94 is disulfide linked with a member of the NKG2 family, namely NKG2A, -B, -C or -E [2,3]. In humans, CD94/NKG2A interacts with complexes of non-classical HLA-E molecules [4,5]. The intracellular domain of NKG2A contains immunoreceptor tyrosine-based inhibition motifs (ITIMs), responsible for transducing inhibitory signals [6]. The other NKG2 members lack ITIMs and are linked to transmembrane proteins, such as DAP10 and DAP12 which contain immunoreceptor tyrosine-based activating motifs and transduce activating signals [7]. CD94/NKG2A is normally expressed on most NK cells and on a small fraction of CD8^+ ^T lymphocytes. The proportion of NK cells bearing the CD94/NKG2A inhibitory receptor decreases in advanced HIV infection [8], in contrast with other inhibitory receptors of the KIR family which are upregulated. It is presently unknown if HIV infection has similar effects on the expression of the CD94/NKG2A inhibitory receptor by CD8^+ ^T cells. A few studies have shown that CD94 expression by CD8^+ ^T cells is increased during HIV infection [9-11] and have led to postulate that increased expression of the CD94/NKG2A inhibitory receptors is one of the mechanisms rendering HIV-specific CD8^+ ^T lymphocytes unable to control HIV-1 infection [12]. Nevertheless, the simultaneous expression of both subunits of the inhibitory receptor on CD8^+ ^T cells has hardly been studied in HIV infection. Costa *et al*. using two-color FACS analysis to study CD3^+ ^NKG2A^+ ^T cells, showed no difference between uninfected controls, long term non progressors or aviraemic subjects under HAART. A slight increase was noted in subjects with active viral replication [13], in contradiction with the downregulation previously observed on NK cells from infected subjects.

**Figure 1:**
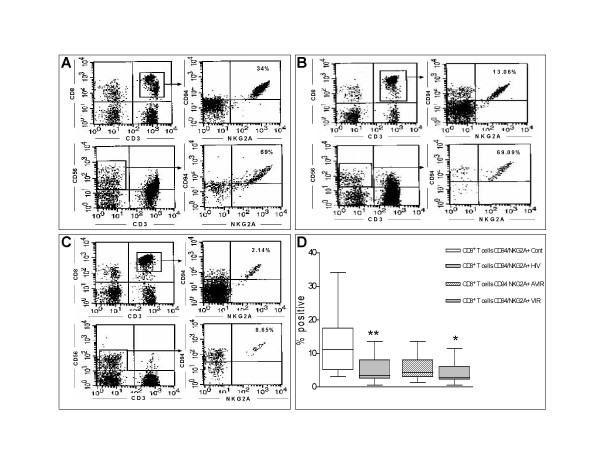
CD94/NKG2A expression in CD8^+ ^T lymphocytes and NK cells from healthy donors and HIV-1 infected patients. Flow cytometry analysis was performed on 100 μl of peripheral blood from **A**: healthy controls, **B**: avireamic HIV-1 infected patients, **C: **vireamic HIV infected patient, using fluorescent conjugated antibodies to CD3, CD8, CD56, CD94, and NKG2A. Analysis was performed on gated cells. **D: **Comparison of CD94/NKG2A expression in CD8^+ ^T lymphocytes from healthy controls (open bar; n = 10), HIV-1 infected patients (vertical hatched bar; n = 46), avireamic HIV-1 infected patients (oblique hatched bar; n = 23) and vireamic HIV-1 infected patients (horizontal hatched bar; n = 23). Data represent the mean ± SEM of each group. The Mann-Whitney test was used to calculate significant differences between the different groups. **p < 0,01 significance of difference of CD94/NKG2A expression in CD8^+ ^T cells between healthy controls vs HIV-1 infected patients; *p < 0,05 significance of difference of CD94/NKG2A expression in CD8^+ ^T cells between HIV-1 infected avireamic patients vs vireamic patients.

In the present study, we used four-colour FACS to investigate the expression of CD94/NKG2A and CD94/NKG2C on CD3^+ ^CD8^+ ^T lymphocytes and NK cells from HIV-1 infected patients, and its relationship with HIV-1 viraemia. Immunostaining was performed with fluorochrome-conjugated antibodies in 100 μl of peripheral blood from HIV-1 infected patients. Participants included 46 HIV-1 infected patients (23 viraemic and 23 aviraemic) and 10 healthy age-matched controls. The cells were analyzed on FACSvantage with CellQuest software (BD Biosciences). Flow Cytometry analysis was performed using fluorescence-conjugated antibodies to CD3, CD8, CD56, CD94, NKG2A and NKG2C. The Mann-Whitney test was used to compare the proportion of cells expressing each heterodimer between the three different groups of subjects (i.e. HIV infected viraemic, aviraemic and non infected controls). Figure 1 (1A, 1B and 1C) shows a representative dot plot of the CD94/NKG2A expression by CD8^+ ^T lymphocytes and NK cells from healthy and HIV-1 infected controls (viraemic and aviraemic). There was a dramatic decline in the proportion of CD8^+ ^T cells expressing the CD94/NKG2A heterodimer in HIV-infected patients in comparison with uninfected controls (mean ± SEM, 4.91 ± 0.49% n = 46 vs. 17.93 ± 3.26% n = 10 p < 0.0001) (Figure 1D). Interestingly, the decrease of CD94/NKG2A expression was more pronounced in patients with detected viral load than in patients with less than 50 copies/ml (mean ± SEM, 4.15 ± 0.65% n = 23 vs. 5.68 ± 0.7190 n = 23; p = 0.0379). Similarly, the proportion of CD8^+ ^T cells expressing the CD94/NKG2C was lower in HIV infected patients than in controls but the difference was not statistically significant (mean ± SEM, 1.73 ± 0.59% n = 16 vs. 5.45 ± 2.25 % n = 10, p = 0.1626) (data not shown).

In HIV infected patients, there was a weak but significant correlation between the proportion of CD8^+ ^T lymphocytes and NK cells expressing the CD94/NKG2A heterodimer (r^2 ^= 0,09184; p = 0.0406) and the proportion of NK cells expressing the inhibitory receptor tended to be lower in viraemic patients than in subjects with less than 50 copies/ml. (mean ± SEM, 43.11 ± 5.67% vs. 56.05 ± 4.67%; p = 0.019). There was no correlation of the expression of the inhibitory receptor with absolute or relative CD4 counts (data not shown).

In summary, we observed a downregulation of CD94/NKG2A on CD8^+ ^T cells in HIV infection, in accordance with what was previously described for NK cells. The mechanisms linking viral replication with downregulation of the inhibitory CD94/NKG2A receptor remains obscure. Upregulation of CD94/NKG2A has previously been observed in various animal models of viral and bacterial infections [14] and in chronic antigenic stimulation [15]. Loss of CD94/NKG2A might correspond to the terminal differentiation which occurs in a large fraction of CD8^+ ^T cells during HIV infection. Indeed, recent observations made in an experimental model of persistent polyoma virus infections suggest that CD94/NKG2A CD8^+ ^T lymphocytes might constitute a less differentiated subset of CD8^+ ^T cells and maintain a higher proliferative potential and capacity to secrete IL-2 [16]. Whatever is the mechanism involved, the loss of CD94/NKG2A in HIV infection could also contribute to the polyclonal activation which characterizes HIV infection.
